# Teaching Bioinformatics in Concert

**DOI:** 10.1371/journal.pcbi.1003896

**Published:** 2014-11-20

**Authors:** Anya L. Goodman, Alex Dekhtyar

**Affiliations:** 1 Department of Chemistry and Biochemistry, California Polytechnic State University, San Luis Obispo, California, United States of America; 2 Department of Computer Science, California Polytechnic State University, San Luis Obispo, California, United States of America; University of British Columbia, Canada

## Abstract

Can biology students without programming skills solve problems that require computational solutions? They can if they learn to cooperate effectively with computer science students. The goal of the in-concert teaching approach is to introduce biology students to computational thinking by engaging them in collaborative projects structured around the software development process. Our approach emphasizes development of interdisciplinary communication and collaboration skills for both life science and computer science students.

## Introduction

Development of computational skills is recognized as an important goal for life science students [1]–[6]; however, current biology and biochemistry curricula at most institutions cannot easily accommodate additional courses in computing sciences. Is it possible to teach computational skills to biologists in a single course? In 2009, Pevzner and Shamir posed this question as a pedagogical challenge: “How should the research and education community design a bioinformatics course that (i) assumes few computational prerequisites, (ii) assumes no knowledge of programming, and (iii) instills in students a meaningful understanding of computational ideas and ensures that they are able to apply them?” [2]

There are two approaches to addressing this challenge. The first approach involves building an introductory programming course into a bioinformatics course, engaging students in the entire process of computational problem solving: problem analysis, design, implementation, and evaluation of the solution. This requires teaching students a programming language (typically Perl or Python) as the means of expressing their solutions. The second approach is to focus on a specific aspect in the problem-solving process, working with students on developing a subset of skills.

Each approach has its own advantages and drawbacks. With the first approach, the students can go through the entire problem-solving process, but the scope of problems they can solve in the confines of a single course is limited. One example of successful implementation of this approach is described by Libeskind-Hadas and Bush [7]. This approach is best suited for curricula that expose students to bioinformatics early and provide subsequent opportunities to advance students' computational skills through additional courses. With the second approach, students go through the specific stages of the problem-solving process working on more complex problems, but they cannot complete the entire problem-solving process on their own. We describe in this essay our initial attempts to implement the second approach and to introduce computational thinking to biology students in a course that does not require any programming from these students.

Can there be computational thinking without programming? Yes! There has been a broad consensus among computer science education researchers and practitioners that the term “computational thinking” is distinct from the term “programming” [8]–[11]. Programming is just one part of the software development process that can be roughly divided into four stages: analysis/requirements, design, implementation (aka programming), and evaluation. The first and the last stages do not require any knowledge of programming languages but rely on solid understanding of the problem that needs to be solved. In our course for life science students, we concentrated on these two stages. The first stage requires the skill recently defined as one of the core competencies for bioinformatics: “an ability to analyze a problem and identify and define the computing requirements appropriate to its solution” [6].

Since we limit the goals of the life science students to analyzing problems, writing program requirements, and evaluating computational solutions and software systems, we need to provide the missing pieces (program design and implementation) in order to complete the problem-solving process. In our course design, we bring together students from two distinct but interconnected courses: Bioinformatics Applications (life science curriculum) and Bioinformatics Algorithms (computer science curriculum). Students in each course are juniors and seniors who have already attained introductory or intermediate skills in their respective disciplines. They attend separate lectures focused on discipline-specific content and then collaborate in the laboratory to build software for solving biological problems.

Is this approach consistent with development of computational thinking? The definition developed by Wing with input from Ato, Cuny, and Snyder refers to computational thinking as “the thought processes involved in formulating a problem and expressing its solution(s) in such a way that a computer—human or machine—can effectively carry out” [12]. Our approach separates the two core components of the definition from each other. “Formulating a problem” is carried out by the life science students, while “expressing the solution” is the job of the computer science students. By separating the two components and stressing only one for each group of students, we are able to significantly increase the complexity of problems that our multidisciplinary student teams can solve. In addition, students practice collaboration and communication skills. Below, we discuss the key features of our approach.

## In-Concert Teaching

In-concert teaching is the approach of teaching two distinct courses in a concerted way. The courses include separate discipline-specific lectures and a shared laboratory component. Each course is taught by the instructor from its respective field and targets distinct audiences of students, but the course materials are developed by two instructors jointly in a coordinated way. Students from both classes form interdisciplinary teams for the duration of the course and work together on laboratory assignments, contributing their discipline-specific knowledge and skills. We call this approach “in-concert” teaching to emphasize the concerted efforts of students and instructors from different disciplines who are working towards accomplishing a common goal [13].

## Distinct and Shared Learning Objectives

Our approach recognizes distinct goals of the life science (BIO) and computer science (CS) courses. We identified distinct, discipline-specific learning objectives, as well as those that are shared. CS students need to learn algorithms and use them for practical problem-solving, while BIO students need to learn to use bioinformatics tools in research. The discipline-specific learning objectives do not require interactions with experts from another discipline, but we believe that cross-disciplinary interactions promote and facilitate student learning within each discipline. In-concert teaching allowed us to expand the list of learning objectives for each group of students to include interdisciplinary collaboration and communication. CS students learn to work with clients who are not programmers, and by the end of the course, they should be able to

1. elicit requirements for new programs,
2. communicate during the software development process to make sure the software meets the needs of the clients, and
3. maintain/modify the delivered software.

Biology students should be able to

1. convert a biological question into a computational one,
2. write program requirements describing the function of the software needed to answer the question, and
3. design test cases to verify that the program developed by their CS partners is working correctly.

Both groups also have shared learning objectives:

1. communicate relevant discipline-specific issues to their partners and
2. cooperate effectively with colleagues within and outside their own discipline on a project.

## Research Problems That Require Collaborative Effort

To create the need for collaboration, instructors of in-concert courses need to identify problems that align with the learning objectives and fall within the scope of each course. The problems have to be complex and interdisciplinary, so that neither group of students can solve them on their own, but also be amenable to analysis by undergraduate students in a fairly short amount of time. Based on our participation in the Genomics Education Partnership (GEP, http://gep.wustl.edu, [14]–[16]), we chose problems related to annotation and comparative analysis of fruit fly genomes. In a set of joint lab assignments distributed over a ten-week quarter, students studied primarily heterochromatic dot/fourth chromosome and a euchromatic region on chromosome 3L. They compared the two regions based on guanine-cytosine (GC) content, gene characteristics, and repetitive sequences. They also developed a program for manipulating GEP data and checking the quality of student-submitted annotations. BIO students also worked on genome annotation projects, which required the following research skills: gathering evidence using bioinformatics tools, analyzing data, and formulating conclusions. In contrast to annotation, comparative genome analysis problems were intentionally poorly defined (more open ended) and gave students an opportunity to practice a complementary set of research skills: defining research questions and developing new tools for answering these questions.

The challenge of comparative genome analysis aligned well with the core content of the CS course, which included simple DNA analysis techniques and measures (GC content, codon bias, and gene content), string comparison (longest common substring, repeat detection, and palindrome discovery), and local and global alignment. CS students built research tools that required the implementation of the data structures, algorithms, and techniques studied in class while also tailoring their implementation to the actual specifics of the problems.

## Interdependent Roles and Peer Instruction

**Figure 1:**
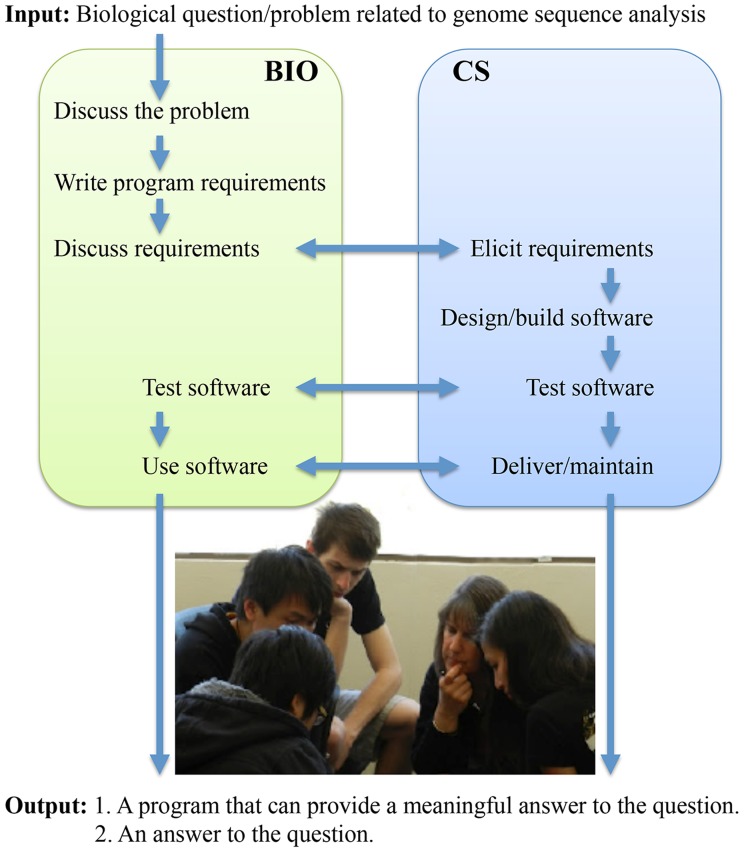
In-concert teaching approach: Clearly defined and interdependent student roles in the joint laboratory are built around software development process.

Recognizing the distinct learning objectives for members of the interdisciplinary teams, we structured teamwork around the software development process and explained to students their distinct and interdependent roles (Figure 1). Biology students worked in groups of two or three to discuss a problem and write a formal program requirements document specifying input, output, and processing needs. During the joint laboratory, BIO students presented these documents to their CS partners and discussed them in detail. While CS students built software, BIO students prepared test cases for evaluation of the software. BIO and CS students worked together on testing the software. Ultimately, biology students were responsible for answering the original question and compiling data from several lab assignments into a final research paper, while CS students supported the delivered software for the remainder of the quarter, providing, upon request, bug fixes, as well as improvements to the tools.

Cross-disciplinary peer instruction was an important component of the course, and we provided students with many opportunities to teach each other. The instruction of CS students in biology was done mainly by their biology partners. Aside from an introductory lecture taught by the BIO course instructor in the CS class, the life science students decided what information was relevant and how to explain concepts of molecular biology to colleagues from a different field. In the BIO class, the CS instructor introduced stages of the software engineering process and explained the role of BIO students in the process, focusing on the development of program requirements. During software testing, which resembles troubleshooting experimental procedures, BIO and CS students learned from each other by contributing different types of tests. BIO students designed small-scale tests and provided expected results based on their knowledge of real data and existing software.

## Student Perception of Working on the Interdisciplinary Teams

We implemented in-concert teaching in our bioinformatics courses in the spring quarter of 2012 (24 BIO and 35 CS students) and 2013 (23 BIO and 26 CS students; course syllabi are provided as Text S1). BIO students included mostly junior- and senior-level students majoring in molecular and cell biology, biochemistry, agriculture, and biomedical engineering. Some of the BIO students completed a prior course in statistics, and only one student in 2013 had programming experience. Most students taking the CS course were in their senior year and came with a considerable computer science background that included the introductory CS sequence (taught in C and Java), data structures, systems programming, and algorithms. Each course included three hours of lecture and three hours of lab per week. The lectures were discipline-specific and were taught separately. The lab sessions took place in two adjacent computer classrooms where students spent most of their time working on joint activities.

We asked students to share their experiences and perceptions of our courses via a voluntary exit survey. Selected quotes in Table 1 are representative examples of student responses to questions regarding teamwork. Communication was listed most prominently both as the benefit and the challenge of working across disciplinary boundaries. When students were asked what they liked the most about the course, the majority of responses fit into three categories: (1) working with partners, (2) working on meaningful projects/real research, and (3) learning specific content from their own discipline. For CS students, the most frequently mentioned category was “working with partners” (15/28 or 53% in 2012, 10/18 or 55% in 2013), while for BIO students, the responses were fairly equally distributed between the three categories (∼30% for each category). We also asked students about challenges and changes they could suggest for the course. These are discussed below. Overall, exit surveys and informal discussions with students after the course suggest that students perceive our bioinformatics courses taught in concert as an extremely challenging but equally worthy experience.

**Table 1:** Selected student responses to questions about cross-disciplinary teamwork from a voluntary exit survey.

Question	Examples of BIO Answer	Examples of CS Answer
What were the benefits of working with [partners from the other discipline]?	“Learning how differently we think from each other and how to communicate more effectively.”	“Having real, nontechnical customers helps you understand the nature of tasks outside the classroom: collaborating with nonsoftware people is unique, their needs are sometimes ambiguous, and there's a frightening but eye-opening reliance on them for domain-specific information. You also get to learn about an interesting field of study that you may have never considered working in before.”
	“Learned to communicate more effectively with people who don't have as [much] of a background in science as we do. We were more able to appreciate what CS people do, and it was fun to work together designing software.”	
What were the challenges/drawbacks of working with [partners from the other discipline]?	“Really the same as the benefits…differences in background knowledge and communication.”	“They struggled tremendously to convey what they wanted the software to do. A lot of the time, also, they didn't have a clear idea of the data they were looking for. Because of that, we were often lost in our job as programmers.…when doing implementation, we usually had to guess their eventual needs or grill them for better details—their written specifications were never enough. This added extra time pressure too, because we'd spend a substantial portion of each lab attempting to work those things out…”
	“When…program functions were not working as expected, that was frustrating. It required patience for all to discuss as a group what was incorrect+brainstorm why.”	
	“They did not know what we wanted, and we did not know what they could do.”	
How did you overcome the challenges?	“Learning to speak up when something is not correct on either side of the team and trying to teach each other bits of background info.”	“We overcame the challenges by talking about the problem and teaching each other things that the other majors did not know…”
	“We had to discuss frequently and rewind our explanations until they made sense.”	

## Challenges

The main pedagogical challenges of the in-concert teaching model are (a) the need to align the content of the two courses and to create a meaningful interdisciplinary experience through the shared laboratory assignments and (b) the need for students to function as experts in their discipline on an interdisciplinary team. We addressed the first challenge by careful planning and preparation, which resulted in developing syllabi for both classes that linked specific lecture topics to laboratory assignments.

The second set of challenges is more difficult to overcome. No single member of the interdisciplinary team has sufficient knowledge/skills to complete a joint lab assignment independently. Rather, the assignments rely on the ability of students to understand “their” parts of the problem and contribute their expertise to the solution (Figure 1). BIO students are expected to understand the nature of the assignment. CS students are expected to be proficient software developers who understand the software requirements for each assignment and build the software. In addition, team members have to get information efficiently across disciplinary boundaries: biologists must put together requirements that CS students can use, while CS students must train biologists in how to use the software. We are addressing this set of challenges by tightening course prerequisites to ensure that only students with appropriate expertise in their discipline are enrolled and by adjusting the lab workflow to require more interdisciplinary interactions.

Finally, assessment presents a major challenge for any educational innovation. It is particularly difficult for us because, to our knowledge, assessment tools for measuring computational thinking skills independently of programming skills are not available. We do use student artifacts (requirements and code) to assess the success of each team. In grading the labs, both artifacts were evaluated, and separate scores were assigned to each. The requirements document score evaluates how well BIO students understood the initial problem they were asked to solve and how well they were able to translate their understanding into a software specification. The software score evaluates the entirety of the process concentrating on the work of CS students. In the 2013 version of the courses, BIO students could request further improvements in the programs throughout the quarter; we collected and evaluated all requests and the modified programs. We are able to assess teams' success in the software development process, and preliminary results of this assessment are described in [13]. The next challenge is to develop tools for assessing computational thinking skills of individual students without relying on the use of any programming language.

## Conclusions

As students advance through their education, they focus more and more on their specific discipline and rarely interact with students in other disciplines in a professional capacity. In most endeavors outside of academia, professionals rarely work in isolation. Anytime a diverse group of professionals is presented with a goal, their success depends on their ability to cooperate effectively across disciplinary boundaries. While this skill is often learned by trial and error on the job, it can also be learned in the classroom. The difference in our approach is that we use a well-established process (software engineering lifecycle) and specifically defined interdependent roles for students in different disciplines to structure collaborative work. We believe that in addition to acquiring communication skills, students deepen knowledge in their own discipline by acting in the roles of experts. In addition, life sciences students are exposed to computational thinking related to requirements specification and software evaluation. The key to our approach is instructor collaboration and identification of a suitable problem: one that is relevant to the learning objectives of each group of students, requires expertise from multiple disciplines, and cannot be solved by either group on their own.

## Acknowledgments

We thank Sally Elgin, Wilson Leung, and the members of the GEP community for discussions, support, and research opportunities for us and our students. We thank Chris Kitts and Michael Black for feedback on the manuscript. We thank Cal Poly Computer Science department for providing facilities to accommodate the classes and our enthusiastic and dedicated teaching assistants Aldrin Montana (2012, 2013), Jan Soliman (2013), and Ryan Verdon (2013).

## Supporting Information

Text S1 **Syllabi from the courses taught in 2013.**
(PDF)
