# Supplementary material for: Teaching Bioinformatics in Concert
Source: PLoS Comput Biol. 2014 Nov 20;10(11):e1003896. doi: 10.1371/journal.pcbi.1003896 (PMC4238947; doi:10.1371/journal.pcbi.1003896)
Supplement: Text S1 — Syllabi from the courses taught in 2013. (PDF) [file pcbi.1003896.s001.pdf]

CHEM441 - BIOINFORMATICS APPLICATIONS
collaboration with CSC448 Bioinformatics Algorithms
Spring 2013

**Lecture:**	Tue, Thur 3:10-4:30	14-302
**Laboratory:**	Tue, Thur 4:40-6	14-302 and 14-301

**Instructor**	**Contact Information**	**Office Hours**
Anya Goodman	Dept. of Chemistry and Biochemistry<br>Office: 25-222 Tel: 756-1666<br>agoodman@calpoly.edu	Tue, Thur 1:10-2:50<br>Mon –floating,<br>e-mail to schedule

COURSE DESCRIPTION

From catalog: Introduction to new problems in molecular biology and current computer applications for genetic database analyses. Use of software for: nucleic acid, genome and protein sequence analysis; genetic databases, database tools; industrial applications in bioinformatics; ethical and societal concerns. 3 lectures, 1 laboratory. Prerequisite: Junior standing; BIO 161 or BIO 303. Recommended: BIO 302 or BIO 303 or BIO 351 or CHEM 373.

COURSE LEARNING OUTCOMES

By the end of the quarter, students should be able to

1. Define, explain and use appropriately terminology and concepts related to genomics, central dogma of molecular biology and molecular evolution.
2. Chose appropriate web-based tools to find information about specific genes, proteins and genomes.
3. Compare two or more nucleotide or protein sequences using BLAST and make inferences regarding molecular evolution and function.
4. Analyze and synthesize information from various databases and BLAST experiments to predict novel gene structure and function.
5. Describe the steps involved in software development process and the role of a client/researcher.
6. Convert a biological question into a computational one and create program requirements for new software.
7. Contribute to software development process by creating testing plan and test cases.
8. Effectively cooperate and communicate with colleagues in life sciences and computer sciences to accomplish a research task.
9. Communicate clearly results of investigation in oral and written form.
10. Describe nature of science, scientific method and its application to genomic research.

ASSESSMENT OF YOUR LEARNING

Your grade in this course will be determined based on the following criteria:

- 35 % joint projects with CSC448: program requirements, genome analysis, paper
- 25 % Genome annotation: tutorials, progress reports, final annotation report
- 20 % Final exam
- 10 % Midterm
- 5% Presentation
- 5 % Attendance, professional conduct, team work: peer evaluation of team members; instructor's observations (see "Attendance" below)

Letter grades will be assigned following roughly 90%, 80%, 70% etc. cut offs for A, B, C etc. respectively.

LEARN-BY-DOING STUDIO FORMAT

This is a "learn-by-doing" course. Our emphasis is on doing research using bioinformatics tools and developing some of these tools. We will have a few formal lectures; these mainly serve to support the laboratory – prepare you for performing specific tasks and discuss what we learn in lab. We will use lecture time for lectures, practice, discussions of lab assignments (program requirements), presentation of lab results (analysis), discussion of our research questions, discussion of homework assignments, quizzes and midterm. The lab time will be used for interactions with CS students (to discuss the goals, divide up the tasks, obtain input data, test programs, obtain real data, analyze real data), gene annotation, and presentation/ discussion of lab results. You will be expected to learn a lot on your own outside of class using resources on the web.

HOMEWORK ASSIGNMENTS

Substantial amount of homework is expected in this course. My assumption is that you have a great capacity for learning on your own and will do it with gentle guidance and blunt feedback from me. For a 4 unit class, expect to put in 8-12 hour per week outside of class. Homework includes (but is not limited to) writing program requirements for each lab, preparing testing plan and test cases for each lab, annotating genes, completing worksheets/ PolyLearn assignments, reading and/or viewing web tutorials. In some cases, you will need to bring a hard copy of your assignment to class and discuss it with your classmates.

TEAM WORK

Many of the assignments in this class will involve team work. Ability to work on a team is an important professional and life skill. In addition, research shows that it can greatly enhance your learning experience. Please, talk to me if you have any concerns or encounter any problems, so we can figure out how to optimize your team's performance and make this a good experience for you.

To facilitate information sharing within each team, we will be using Piazza. Once the teams are organized, each team will have its own Piazza group, and will be able to share documentation/software with the teammates. The address for the Piazza web page for CSC 448/CHEM 441 is:
https://piazza.com/class\#spring2013/csc448bio441

ATTENDANCE

Attendance in this course is very important. Your team's success depends on you! If you have to miss a class or part of a class for one of the reasons recognized by Cal Poly (see http://www.academicprograms.calpoly.edu/content/academicpolicies/class-attendance), talk to me in advance (if possible) about an "excused" absence, and we can try to make arrangements with your

team to minimize the damage. One unexcused absence will result in the penalty of 5% of the final grade; every subsequent unexcused absence will result in the loss of 10% or more of the course grade. If/when your team is looking for you during scheduled class time – you are considered absent.

EXAMS

Midterm and the final exam allow for individual assessment of students' mastery of skills, concepts and procedures. Midterm will assess mastery of annotation procedures (tutorials) and basic concepts of molecular biology and software development. Final exam will focus on writing program requirements, testing, comparative genomics, and annotation (our research project). The questions will be short answer, draw a diagram, fill in the blank.

REFLECTION AND EDUCATION RESEARCH

To help improve this course, I will ask you to reflect on your experiences, assignments, and group dynamics. In addition, you will be asked to participate in several educational research assessments (pre- and post-course surveys and quizzes) to help improve instructional design and activities. Participation in these is voluntary.

RESEARCH PROJECT

We will work on a research project in collaboration with Genomics Education Partnership (GEP, www.gep.wustl.edu) studying a recently sequenced genome. Our research is focused on comparative genomics and genome annotation. **Our research goals are:**

1. To compare genome structure of *a new genome* to other closely related Drosophila species and/or compare large regions of the same genome to each other (chr. 4 vs. chr.3 comparison).
2. To find repeats, protein coding genes and non-coding functional elements in a "finished" genome sequence (annotation).

The course assignments can be divided in two parts, but we will work on the two parts concurrently:

I. Lab assignments carried out jointly with CSC448 students:

BIO/CHEM students will write program requirements, test the programs written by CS students and analyze data to answer biological questions.

The graded products for each lab are:

1. Program requirements document
2. Program test cases and testing report
3. Data analysis, compiled at the end of the quarter into a final team paper.

II. Genome annotation:

A. Practice annotation (use previously completed annotation projects, known answers)

B. Teams work on new annotation projects. Progress reports and short presentations will be done during lab/lecture.

C. Quality control: each team will check and submit another team's projects.

Final product will be a set of 4 files for each contig:

1. Annotation report file (Word file with screen shots)
2. contigX.fasta (multiple sequence FASTA file with predicted CDS of all isoforms for all genes)
3. contigX.pep (multiple sequence FASTA text file with predicted amino acid sequence of all isoforms for all genes)
4. contigX.gff (GFF formatted description of all isoforms for all genes).

Tentative COURSE CALENDAR (for detailed/updated plan see PolyLearn)

Wk	Date	3:10-4:00 Lecture/Annotation	4:00-4:30 Lab prep	4:40-6 Lab with CSC448 and annotation
1	Apr 2	Course overview/syllabus<br>GEP Assessment -QUIZ	GEP survey	*Software development lecture by Dr. Dekhtyar*
	4	Critical Thinking test (CAT)	Intro to bioinformatics	L1. Introductory activity
2	Apr 9	Annotation: UCSC genome browser	L2-1 discussion: Requirements	**L2-1.GC Content**
	11	Annotation: BLAST<br>Research project intro	L2-2 Discussion: Reqs and testing	**L2-2. GC content** requirements
3	Apr 16	Annotation: BLAST, Gene Finder, Gene Model checker	L3 Intro	L2-2 GC content testing
	18	Annotation: BLAST	L3 Requirements	**L3 Gene content** requirements Annotation
4	Apr 23	Practice contig<br>Software life cycle lecture	*Software life cycle lecture (Dekhtyar)*	L3 testing, debugging
	25	Practice contig	L4 Intro	L3 testing, debugging
5	Apr 30	**MIDTERM**	L4 Requirements	**L4 Repeat finding**
	2	Midterm (?)<br>Annotation project assignment	Annotation	*Software testing lecture by Dr. Dekhtyar*
6	May 7	Research project overview, MicroRNA	L5 Intro	L4 Testing
	9	Sequencing data and databases	L5 Requirements	**Lab 5 MicroRNA finding**
7	May 14	Sequence comparison	L5 Testing	L5 testing
	16	Sequence comparison	L6 Intro	L5 testing
8	May 21	Multiple sequence alignments and trees	L6 Requirements	**L6 Contig Merger and Annotation QC**
	23	Multiple sequence alignments and trees	L6 Testing	L6 testing
9	May28	No class – Mon schedule		
	30	12 genomes papers	Sci Method activity	L6 testing
10	June 4	CAT test (1 hr)<br>Student Presentations	Student Presentations	Student Presentations
	6	GEP Quiz/Survey/ Student Presentations	Student Presentations	Student Presentations
	June 11	**FINAL EXAM** /exit survey	4:10-7	

CSC 448: Bioinformatics Algorithms
Spring 2013
Course Syllabus

March 31, 2013

Instructor: Alexander Dekhtyar
email: dekhtyar@calpoly.edu
office: 14-215

What	When		Where
Lecture	TR	3:10 – 4:30pm	10-227
Lab	TR	4:40 – 6:00pm	14-302
**Final Exam time**	June 11 (Tuesday)	4:10 - 7:00pm	10-227

Office Hours

	When	Where
Monday	1:10pm - 2:00pm	14-215
Tuesday	2:10pm - 3:00pm	14-215
Wednsday	9:10am - 12:00pm	14-215

Additional appointments can be scheduled by emailing the instructor at *dekhtyar@calpoly.edu*.

Description

This course studies the application of computing techniques to solving problems in the field of bioinformatics and computational molecular biology.

The key learning outcomes for the course are as follows. Upon completion of the course, you will:

- Know the main problems in the field of bioinformatics and computational molecular biology.

- Understand the key algorithms used to solve computational biology and bioinformatics problems
- Model computational biology problems
- Apply algorithmic techniques to solve problems in computational biology and bioinformatics
- Gain experience working on software projects on multidisciplinary teams.

Texbook

The material for this course comes primarily from these two books:

- N.C. Jones, P. Pevzner. *An Introduction to Bioinformatics Algorithms*, MIT Press, 2004, ISBN:978-0-262-10106-6.
- D. Gusfield, *Algorithms on Strings, Trees and Sequences: Computer Science and Computational Biology*, Cambridge University Press, 1st Ed., 1997, ISBN: 978-0521585194.

Topics

No.	Topic	Number of Lectures
1.	Introduction: Biology, Bioinformatics resources	2
2.	Statistical Analysis of DNA	2
3.	Exact String Matching	4
4.	Palindrome Detection	1
5.	Genome Alignment	4
6.	Gene Prediction	2
7.	Clustering	2
8.	Advanced Topics	1-2

Grading

**Interdisciplinary Labs**	50-60%
**Homeworks**	5-10%
**Midterm**	10-20%
**Final Exam**	20-30%

Course Policies

Exams

Content-wise, this is essentially an advanced algorithms course. There course will have a midterm exam and a final exam. The tentative date for the midterm exam is *May 7 (Tuesday) or May 9 (Thursday).*

The date for the final exam is *Tuesday, June 11, 4:10 - 7:00pm.*

Both exams will be written tests designed to test your knowledge of the algorithms and data structures covered in the course.

Labs, CHEM 441

The course is taught *in concert* with CHEM 441, Bioinformatics Applications. The two courses discuss the field of bioinformatics from two different perspectives. CSC 448 is a course targeted at computing sciences majors (CSC, CPE, SE). It will concentrate on algorithms and software design and development for bioinformatics problems. CHEM 441 is targeted as life sciences and biotechnology majors (BIO, BCHM, CHEM, BMED, ANSC). It concentrates on the biological nature of the problems for which computational solutions are required, and on the use bioinformatics software in the field of biology.

Teaching the courses in concert means the following:

- Course content is coordinated between the CSC 448 (Alex Dekhtyar) and CHEM 441 (Anya Goodman) instructors.
- All major hands-on laboratory assignments are shared between the two courses. While the requirements, expectations and deliverables for the assignments will be different, the work on these assignments will be performed by multidisciplinary teams formed jointly out of students from both courses.

Both classes are taught at the same time, with the labs periods being scheduled in nearby labs.

All major labs will be performed in joint teams. Teams will be formed during the first/second week of the classes, and will persist over the course of the quarter. The majority of the lab assignments is designed to contribute to a single overarching course project the students of CHEM 441 will be working on (hence, the team persistance).

Lab time will be split between lab periods designated for joint work between CSC 448 and CHEM 441 students and lab periods designated for independent software development work by the CSC 448 subteams and independent work by CHEM 441 teams.

The major joint labs are:

Lab Number	Lab Topic	Lab Duration
Lab 1	Ice-breaking activities	1 class
Lab 2	Simple DNA analysis	1.5 weeks
Lab 3	Statstical analysis of DNA	1.5 weeks
Lab 4	Search for repeated sequences	1.5 weeks
Lab 5	MicroRNA search	1.5 weeks
Lab 6	Genome Annotation (alignment)	2 weeks
Lab 7	Poster	1 week

Homeworks

Some amount of paper-and-pencil homeworks is expected.

Communication

The class will have an official mailing list. The email address for the mailing list is *csc-448-01-2134@calpoly.edu*. All students enrolled in the class are automatically subscribed to the mailing list (using the email addresss that the CS department has on file).

We will also have a joint mailing list for CSC 448/CHEM 441 students.

I encourage questions during classtime and questions via email. My answers to email questions may be broadcast to the entire class via the mailing list, if the answer may be relevant to everyone (e.g. a correction in a text of a handout, or a clarification of a homework problem), and may also appear on the web page. The questions can also be posted to the mailing list directly. The mailing list will also be used for all annoucements related to the course. It is your responsibility to read your class-related email. Failure to read email posted to the mailing list cannot be used as an excuse in the class.

Web Page

Class web page can be found at

http://www.csc.calpoly.edu/∼dekhtyar/448-Spring2013

Through this page you will be able to access all class handouts including homeworks, lab assignments, project information, lab/project data and lecture notes.

Links to additional information, and notes and announcements will also be posted.

Piazza

To facilitate information sharing within each team, we will be using Piazza. The address for the Piazza web page for CSC 448/CHEM 441 is:

https://piazza.com/class#spring2013/csc448bio441

Once the teams are organized, each team will have its own Piazza group, and will be able to share documentation/software with the teammates.

Academic Integrity

University Policies

Cal Poly's Academic Integrity policies are found at

http://www.academicprograms.calpoly.edu/academicpolicies/Cheating.htm

In particular, these policies define *cheating* as (684.1)

> "*. . . obtaining or attempting to obtain, or aiding another to obtain credit for work, or any improvement in evaluation of performance, by any dishonest or deceptive means. Cheating includes, but is not limited to: lying; copying from another's test or examination; discussion of answers or questions on an examination or test, unless such discussion is specifically authorized by the instructor; taking or receiving copies of an exam without the permission of the instructor; using or displaying notes, "cheat sheets," or other information devices inappropriate to the prescribed test conditions; allowing someone other than the officially enrolled student to represent same.*"

Plagiarism, per University policies is defined as (684.3)

> "... the act of using the ideas or work of another person or persons as if they were one's own without giving proper credit to the source. Such an act is not plagiarism if it is ascertained that the ideas were arrived through independent reasoning or logic or where the thought or idea is common knowledge. Acknowledgement of an original author or source must be made through appropriate references; i.e., quotation marks, footnotes, or commentary."

University policies state (684.2): "Cheating requires an "F" course grade and further attendance in the course is prohibited." (appeal process is also outlined, see the web site above for details.). Plagiarism, per university policies (684.4) can be treated as a form of cheating, although a level of discretion is given to the instructor, allowing the instuctor to determine the causes of plagiarism and effect other means of remedy. It is the obligation of the instructor to inform the student that a penalty is being assessed in such cases.

Course Policies

All homeworks are to be completed by each student **individually**. Lab assignments are to be completed by the appropriate units (individual, pair, group), and no code/solution-sharing between units is permitted. Students are encouraged to discuss class content among themselves but NOT in a manner that constitutes plagiarism and cheating as defined above (e.g., you can solve together a problem from the textbook that had not been assigned in the homework, but you should solve assigned problems individually).
